# Occupational noise exposure and its association with incident hyperglycaemia: a retrospective cohort study

**DOI:** 10.1038/s41598-020-65646-1

**Published:** 2020-05-22

**Authors:** Ta-Yuan Chang, Tzu-Yi Yu, Chiu-Shong Liu, Li-Hao Young, Bo-Ying Bao

**Affiliations:** 1 Department of Occupational Safety and Health, College of Public Health, China Medical University, No. 91, Hsueh-Shih Road, Taichung, 40402 Taiwan Republic of China; 2 Department of Family Medicine, China Medical University Hospital, No. 2, Yuh-Der Road, Taichung, 40447 Taiwan Republic of China; 3 Department of Pharmacy, College of Pharmacy, China Medical University, No. 91, Hsueh-Shih Road, Taichung, 40402 Taiwan Republic of China; 4 Department of Nursing, Asia University, No. 500, Lioufeng Road, Wufeng, Taichung, 41354 Taiwan Republic of China

**Keywords:** Pre-diabetes, Pre-diabetes

## Abstract

Noise pollution is reported to be associated with diabetes, but few studies have elucidated the associations between noise frequency characteristics. We aimed to evaluate the relationships between different noise frequency components and incident hyperglycaemia. An industry-based cohort of 905 volunteers was enrolled and followed up to 2012. Octave-band frequencies of workstation noise and individual noise levels were measured in 2012 to classify subjects’ exposures retrospectively. We applied Cox regression models to estimate the relative risk (RR) of hyperglycaemia. An increased RR for hyperglycaemia of 1.80 (95% confidence interval [CI]: 1.04, 3.10) was found among subjects exposed to ≥ 85 A-weighted decibels (dBA) compared with those exposed to < 70 dBA. The high-exposure groups at frequencies of 31.5, 63, 125, 250, 500, 1000, and 2000 Hz had a significantly higher risk of hyperglycaemia (all p values < 0.050) than the low-exposure groups. A 5-dB increase in noise frequencies at 31.5, 63, 125, 250, 500 Hz, and 1000 Hz was associated with an elevated risk of hyperglycaemia (all p values < 0.050), with the highest value of 1.27 (95% CI: 1.10, 1.47) at 31.5 Hz (p = 0.001). Occupational noise exposure may be associated with an increased incidence of hyperglycaemia, with the highest risk observed at 31.5 Hz.

## Introduction

Growing numbers of field studies have reported an association between occupational noise exposure and cardiovascular disease (CVD) morbidity and mortality^1–10^. In 2019, the World Health Organization (WHO) and the International Labour Organization (ILO) provided a joint methodology (WHO/ILO joint methodology) for estimating the work-related burden of CVD and injury due to workplace noise exposure^11^. Noise exposure is regarded as an environmental stressor through direct (i.e., sleep disturbance) and indirect (i.e., annoyance) pathways that pose adverse health effects. Acute noise exposure may activate the hypothalamus-pituitary-adrenal axis and the sympathetic-adrenal-medulla axis to elevate the levels of stress hormones, including cortisol, catecholamine, adrenalin, and noradrenalin. Repeated and chronic stimuli may cause overproduction of stress hormones (e.g., cortisol) that increase the levels of fatty acids and glucose to partially restore homeostasis. In addition, catecholamines also boost the energy supply by breaking down triacylglycerol. Such increases in stress hormones may lead to pathophysiologic alterations in blood pressure, blood lipids, blood viscosity, and blood glucose, which promote the development of hypertension, arteriosclerosis, and CVD^12–14^.

Because the overproduction of cortisol may inhibit pancreatic insulin secretion and reduce insulin sensitivity in the liver, skeletal muscles, and adipose tissue^14^, it is plausible that long-term noise exposure may produce adverse changes in blood glucose. Noise pollution has been reported to be associated with diabetes in many animal and environmental epidemiological studies; however, few studies have elucidated the relationship between noise frequency characteristics and diabetes. Two animal studies observed that diabetes aggravated noise-induced hearing loss in male Wistar rats^15,16^. Chronic noise exposure has been found to lead to diabetes from changes in the gut microbiota composition and intestinal inflammation in rats^17^ and from the exacerbation of insulin resistance, promoting the manifestation of diabetes in mice^18^. A cross-sectional study also reported that impaired fasting glucose was associated with noise-induced hearing loss among automobile manufacturing workers^19^. A 2014 national survey in the US general population found that self-reported occupational noise exposure was associated with an increased risk of obesity and an elevation of measured body mass index (BMI)^20^. Environmental epidemiological studies have demonstrated that traffic noise exposure is associated with diabetes incidence^21^ and mortality^22^. A cohort study in Europe reported that one interquartile range (IQR) increase in noise exposure (4.2 A-weighted decibels [dBA]) was associated with an elevation of 0.2% (95% confidence interval [CI]: 0.1–0.3%) in fasting glucose^23^. In a systematic review, a 5-dB increase in noise exposure was associated with an increased risk of a 6.0% (95% CI: 3.0–9.0%) increase in diabetes incidence, mainly related to air and road traffic noise^24^. To the best of our knowledge, no studies have investigated the relationship between occupational noise exposure and incident hyperglycaemia. Furthermore, the association between incident hyperglycaemia and different noise frequency characteristics is unknown. Therefore, the purpose of this retrospective study was to elucidate the relationship between occupational noise exposure and the incidence of hyperglycaemia. We also determined whether there were differences in the associations between hyperglycaemia and different noise frequency components.

## Results

### Demographic description of the study population

The demographic characteristics of the three study groups are presented in Table 1. Significant group differences in mean age, employment duration, and systolic blood pressure (SBP) and in the proportions of male sex, high educational level, current smokers, regular exercisers, high working activity, and workers using hearing-protection devices were observed (all p values < 0.050). The high- and medium-exposure groups had significantly higher means of SBP and proportions of male sex, current smokers, and high working activity but lower mean employment duration and proportions of high educational level and regular exercise than the low-exposure group. In addition, workers in the high-exposure group were more likely to use hearing-protection devices than those in the medium- and low-exposure groups (both p values < 0.001).

**Table 1:** Demographic characteristics of the subjects of the study conducted in 2012 in Taichung, Taiwan.

Variable	Noise exposure group						Total subjects (n = 905)	
	High (n = 108)			Medium (n = 433)		Low (n = 364)		
	Mean (SD)	*P* value^c^	*P* value^d^	Mean (SD)	*P* value^c^	Mean (SD)	Mean (SD)	*P* value^a^
Age, years	38.8 (10.8)	0.650	0.007	35.4(7.6)	< 0.001	39.0(7.8)	37.3(8.3)	< 0.001
Employment duration, years	10.1 (9.5)	0.018	0.098	7.3(6.0)	< 0.001	11.6(8.2)	9.4(7.7)	< 0.001
Body mass index, kg/m^2^	24.1 (3.5)	0.786	0.516	24.3(3.7)	0.167	24.0(3.8)	24.2(3.7)	0.372
Systolic blood pressure, mmHg	125.9 (13.8)	0.051	0.679	126.0 (12.3)	< 0.001	123.3 (14.5)	124.9 (13.4)	0.001
Diastolic blood pressure, mmHg	82.9 (10.3)	0.422	0.586	82.2 (9.4)	0.673	82.2 (10.8)	82.3 (10.1)	0.714
Triglyceride level, mg/dL	109.7 (73.0)	0.059	0.135	120.2(77.3)	0.530	124.9(98.7)	120.8(86.2)	0.168
Total cholesterol level, mg/dL	185.4 (35.7)	0.222	0.587	188.9(35.8)	0.376	189.8(34.1)	188.9(35.1)	0.439
	**No. (%)**	***P*** **value**^**e**^	***P*** **value**^**f**^	**No. (%)**	***P*** **value**^**e**^	**No. (%)**	**No. (%)**	***P*** **value**^**b**^
Sex, male	94 (87.0)	< 0.001	0.809	373 (86.1)	< 0.001	259 (71.2)	726 (80.2)	< 0.001
Educational level, >12 years	25 (23.2)	< 0.001	< 0.001	210 (48.5)	<0.001	300 (82.4)	535 (59.1)	< 0.001
Current smoker, yes	42 (38.9)	< 0.001	0.039	124 (28.6)	< 0.001	58 (15.9)	224 (24.8)	< 0.001
Alcohol consumption, yes	17 (15.7)	0.060	0.445	56 (12.9)	0.111	34 (9.3)	107 (11.8)	0.119
Regular exercise, yes	40 (37.0)	0.115	0.330	139 (32.1)	< 0.001	166 (45.6)	345 (38.1)	< 0.001
Hypertension, yes	30 (27.8)	0.413	0.972	121 (27.9)	0.195	87 (23.9)	238 (26.3)	0.405
Family history of diabetes, yes	17 (15.7)	0.195	0.507	80 (18.5)	0.298	78 (21.4)	175 (19.3)	0.346
Working activity, high	40 (37.0)	< 0.001	0.198	190 (43.9)	< 0.001	35 (9.6)	265 (29.3)	< 0.001
Use of hearing-protection devices at work, yes	21 (19.4)	< 0.001	< 0.001	20 (4.6)	0.001	3 (0.8)	44 (4.9)	< 0.001

SD, standard deviation. ^a^Kruskal-Wallis test between the three groups. ^b^Chi-square test between the three groups. ^c^Wilcoxon rank-sum test with the low-exposure group. ^d^Wilcoxon rank-sum test with the medium-exposure group. ^e^Chi-square test with the low-exposure group. ^f^Chi-square test with the medium-exposure group.

### Personal and workstation noise exposure assessment

Supplemental Table S1 shows the correlations between personal exposure, workstation levels, and octave-band frequencies of workplace noise. Personal noise levels correlated significantly with workstation levels and all octave bands (all p values < 0.001), and higher correlations (correlation coefficients > 0.810) were observed at frequencies of 250, 500, and 1000 Hz.

Personal noise levels and octave band analyses of workstation noise pertaining to different groups are presented in Table 2. Significantly higher mean workstation levels and personal noise exposure were observed in the high- and medium-exposure groups than in the low-exposure group (both p values < 0.001). In addition, the high- and medium-exposure groups had significantly higher averages of noise levels at all octave bands than the low-exposure group (all p values < 0.001).

**Table 2:** Means and standard deviations of noise exposure and frequency components for participants measured in 2012 in Taichung, Taiwan.

Variable	Noise exposure group						Total		*P* value
	High		Medium		Low				
	Mean (SD)	Median (IQR)	Mean (SD)	Median (IQR)	Mean (SD)	Median (IQR)	Mean (SD)	Median (IQR)	
Personal level (dBA)	88.2 (2.8)	87.8 (2.5)^b,c^	77.0 (6.5)	79.0 (6.3)^c^	67.7 (3.8)	66.4 (6.6)	74.6 (8.4)	75.2 (15.1)	< 0.001^a^
Workstation level	80.9 (4.4)	80.2 (6.3)^b,c^	72.7 (8.6)	73.4 (9.5)^c^	57.1 (3.1)	55.0 (3.4)	67.4 (10.9)	68.5 (21.6)	< 0.001^a^
(full frequency, dBA)									
31.5 Hz (dB)	35.9 (4.3)	36.2 (5.5)^b,c^	31.2 (5.0)	30.7 (6.9)^c^	25.4 (4.6)	21.7 (7.8)	29.4 (6.0)	29.3 (10.1)	< 0.001^a^
63 Hz (dB)	47.9 (3.8)	47.9 (5.5)^b,c^	42.2 (6.5)	43.7 (7.3)^c^	33.1 (4.8)	30.1 (4.7)	39.2 (7.7)	41.5 (14.7)	< 0.001^a^
125 Hz (dB)	56.0 (5.0)	56.9 (4.4)^b,c^	50.8 (6.6)	51.4 (8.0)^c^	38.6 (5.3)	34.9 (5.7)	46.5 (9.0)	48.2 (15.3)	< 0.001^a^
250 Hz (dB)	63.0 (5.0)	64.1 (4.8)^b,c^	57.8 (7.0)	58.6 (7.1)^c^	45.2 (3.8)	42.5 (4.0)	53.4 (8.9)	53.9 (15.9)	< 0.001^a^
500 Hz (dB)	69.8 (5.1)	68.9 (7.9)^b,c^	63.4 (6.6)	65.2 (6.2)^c^	51.8 (4.1)	48.5 (5.9)	59.5 (8.6)	60.0 (14.9)	< 0.001^a^
1000 Hz (dB)	72.9 (4.1)	72.8 (6.8)^b,c^	65.4 (7.2)	67.3 (7.0)^c^	52.7 (3.9)	49.8 (4.7)	61.2 (9.3)	61.2 (17.1)	< 0.001^a^
2000 Hz (dB)	74.3 (4.2)	74.0 (4.5)^b,c^	65.4 (7.2)	65.8 (8.0)^c^	55.5 (1.6)	55.8 (2.1)	62.5 (8.2)	59.4 (14.8)	< 0.001^a^
4000 Hz (dB)	75.3 (4.5)	75.3 (4.3)^b,c^	66.2 (7.2)	65.7 (9.7)^c^	53.6 (1.4)	54.7 (2.5)	62.2 (9.4)	59.9 (17.6)	< 0.001^a^
8000 Hz (dB)	71.6 (4.5)	70.6 (5.7)^b,c^	63.8 (7.5)	62.3 (10.3)^c^	51.4 (0.8)	52.1 (1.6)	59.7 (9.1)	59.3 (16.7)	< 0.001^a^

dB, decibel; dBA, A-weight decibel; IQR, interquartile range; SD, standard deviation. ^a^Kruskal-Wallis test (p < 0.050) between the three groups. ^b^Wilcoxon rank-sum test (p < 0.050) with the medium-exposure group. ^c^Wilcoxon rank-sum test (p < 0.050) with the low-exposure group.

### Incident hyperglycaemia and associations with occupational noise exposure

The mean fasting blood glucose (FBG) and the relative risk (RR) of hyperglycaemia for the three groups are shown in Table 3. A significant difference in FBG between groups was observed (p = 0.001). Only the high-exposure group had a significantly higher mean FBG than the medium-exposure group (p = 0.004).

**Table 3:** Fasting blood glucose and relative risk of hyperglycaemia in the study group.

Noise exposure group	n	FBG, mg/dl, mean (SD)	HG cases, n	Person-years	Incident rate	Crude RR (95% CI)	*P* value
Low (< 70 dBA)	364	90.9 ± 9.5	47	3149.7	1.49 × 10^−2^	1.00	—
Medium (70–85 dBA)	433	89.4 ± 12.1	51	2695.3	1.89 × 10^−2^	1.36(0.81–2.30)	0.246
High (>= 85 dBA)	108	92.9 ± 13.2^b^	21	965.9	2.17 × 10^−2^	1.44(0.96–2.16)	0.078
		P = 0.001^a^					

CI, confidence interval; FBG, fasting blood glucose; HG, hyperglycaemia; RR, relative risk; SD, standard deviation. ^a^Kruskal-Wallis test between the three groups. ^b^Wilcoxon rank-sum test (p < 0.050) with the medium-exposure group.

Table 4 presents the association between occupational noise exposure and the risk of incident hyperglycaemia. Compared with workers exposed to < 70 dBA, workers exposed to ≥ 85 dBA had an increased RR for hyperglycaemia of 1.80 (95% CI: 1.04, 3.10; p = 0.034) after adjusting for age, sex, triglyceride level, hypertension, family history of diabetes, and the use of hearing-protection devices. An exposure-response association was found between noise exposure and the risk of hyperglycaemia for all three groups (adjusted RR [ARR] = 1.33; 95% CI: 1.02, 1.73; p = 0.033). In a sensitivity analysis, subjects exposed to ≥ 85 dBA had an elevated risk of hyperglycaemia (ARR = 1.60; 95% CI: 0.97, 2.64; p = 0.066) than those exposed to < 85 dBA, but the result was marginally significant. Only sex was found as an effect modifier for the comparisons between the high-exposure and low-exposure groups, as shown in Supplemental Figure S2 (p = 0.020). Women were more susceptible to the increased risk of incident hyperglycaemia than men (ARR = 6.63; 95% CI: 1.99, 22.11; p = 0.002).

**Table 4:** Association between occupational noise exposure and risk of incident hyperglycaemia among participants.

Variable	Model 1^a^			Model 2^b^			Model 3^c^		
	ARR	95% CI	*P* value	ARR	95% CI	*P* value	ARR	95% CI	*P* value
**Noise exposure group**									
Low (< 70 dBA)	1.00	Referent	—	1.00	Referent	—	1.00	Referent	—
Medium (70–85 dBA)	1.47	0.98, 2.20	0.065	1.38	0.92, 2.07	0.120	1.29	0.85, 1.96	0.240
High (>= 85 dBA)	1.63	0.95, 2.79	0.076	1.82	1.06, 3.11	0.030	1.80	1.04, 3.10	0.034
**Use of hearing-protection devices**									
Yes versus No	0.36	0.13, 1.01	0.053	0.36	0.13, 1.02	0.053	0.36	0.13, 1.02	0.054
**Hypertension**									
Yes versus No				1.65	1.13, 2.42	0.009	1.66	1.13, 2.43	0.009
**Triglyceride level, mg/dL**									
≥ 99 versus < 99				1.58	1.07, 2.34	0.023	1.60	1.06, 2.40	0.024
**Family history of diabetes**									
Yes versus No							1.12	0.73, 1.71	0.613

ARR, adjusted relative risk; CI, confidence interval; dBA, A-weighted decibel. ^a^Cox regression model adjusted for the use of hearing-protection devices. ^b^Cox regression model adjusted for the use of hearing-protection devices, hypertension, and triglyceride level. ^c^Cox regression model adjusted for age, sex, triglyceride level, hypertension, family history of diabetes, and the use of hearing-protection devices.

### Noise frequency characteristics and incident hyperglycaemia

**Figure 1:**
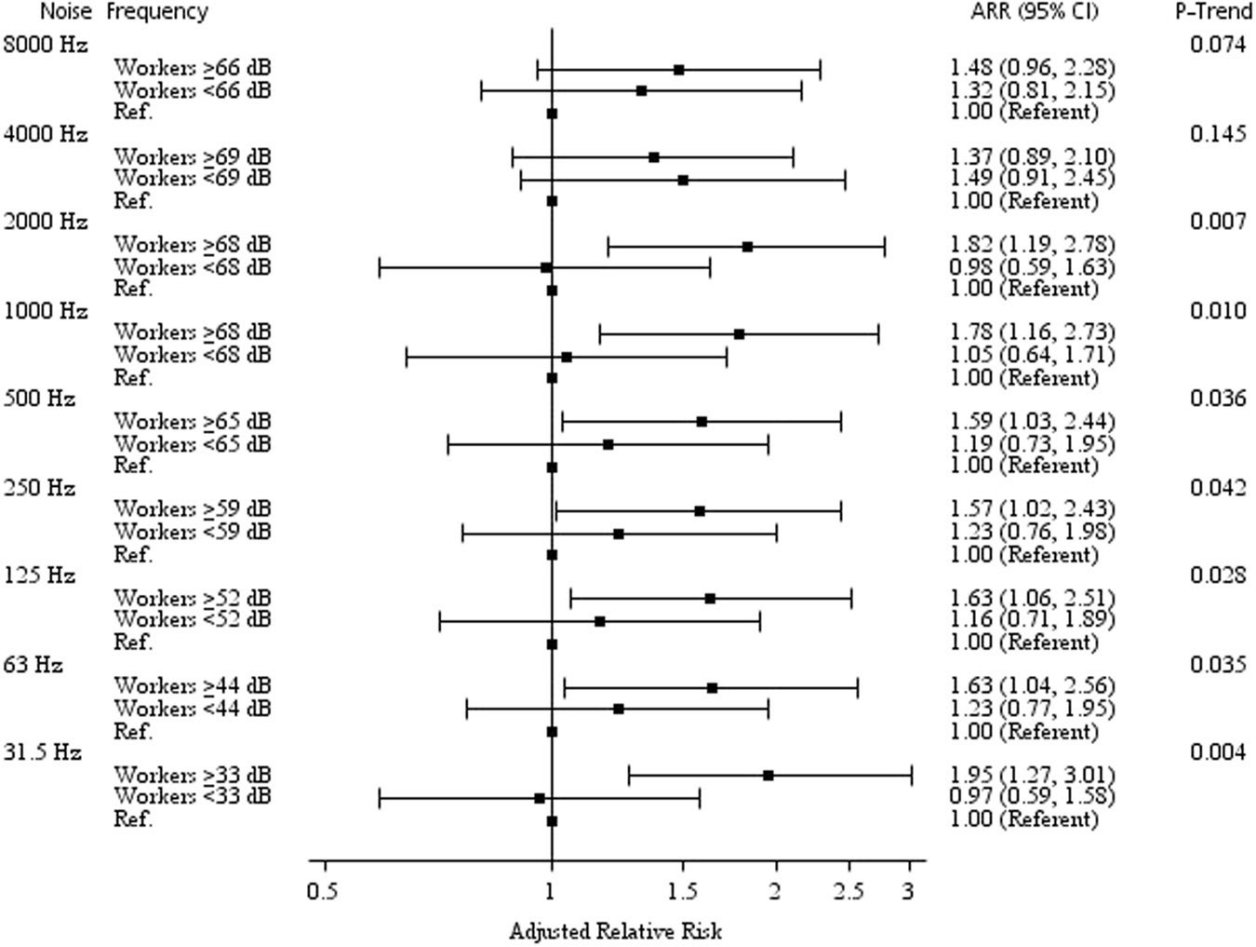
Adjusted relative risk (ARR)^a^ of incident hyperglycaemia according to the octave-band frequencies of occupational noise exposure for participants. ARR, adjusted relative risk; CI, confidence interval; dB, decibel; Ref, reference (i.e., officers). ^a^Cox regression model adjusted for age, sex, triglyceride level, hypertension, family history of diabetes, and the use of hearing-protection devices.

Figure 1 shows the risk of incident hyperglycaemia according to the octave-band frequencies of workstation noise by group. Compared with the low-exposure groups, the high-exposure groups at frequencies of 31.5, 63, 125, 250, 500, 1000, and 2000 Hz had significantly increased risks of incident hyperglycaemia (all p values < 0.050). The strongest association was observed at 31.5 Hz. Compared with those exposed to 25.4 ± 4.6 dB at 31.5 Hz, subjects exposed to 36.7 ± 3.1 dB at the same frequency had an increased RR for hyperglycaemia of 1.95 (95% CI: 1.27, 3.01; p = 0.002). Significant exposure-response trends were found at 31.5 Hz (ARR = 1.39; 95% CI: 1.11, 1.74; p = 0.004), 63 Hz (ARR = 1.28; 95% CI: 1.02, 1.60; p = 0.035), 125 Hz (ARR = 1.28; 95% CI: 1.03, 1.59; p = 0.028), 250 Hz (ARR = 1.25; 95% CI: 1.01, 1.56; p = 0.042), 500 Hz (ARR = 1.26; 95% CI: 1.02, 1.56; p = 0.036), 1000 Hz (ARR = 1.34; 95% CI: 1.07, 1.66; p = 0.010), and 2000 Hz (ARR = 1.35, 95% CI: 1.09, 1.68; p = 0.007). Because all results (i.e., 31.5 Hz [p = 0.666], 63 Hz [p = 0.512], 125 Hz [p = 0.580], 250 Hz [p = 0.551], 500 Hz [p = 0.421], 1000 Hz [p = 0.729], 2000 Hz [p = 0.681], 4000 Hz [p = 0.292], and 8000 Hz [p = 0.319]) had p values > 0.050, no interactions of sound intensity and frequency components could be identified.

**Figure 2:**
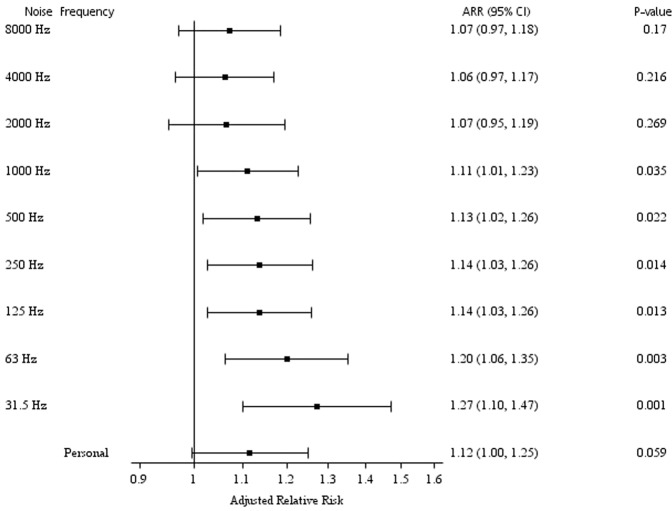
Adjusted relative risk (ARR)^a^ of incident hyperglycaemia according to 5-dBA increase in personal noise levels and 5-dB increase in octave-band frequencies among participants. ARR, adjusted relative risk; CI, confidence interval; dB, decibel; dBA, A-weighted decibel. ^a^Cox regression model adjusted for age, sex, triglyceride level, hypertension, family history of diabetes, and the use of hearing-protection devices.

The risk of incident hyperglycaemia according to the 5-dBA increase in personal noise levels and the 5-dB increase in the octave-band frequencies is shown in Figure 2. Five-dB increases in workstation noise levels at frequencies of 31.5, 63, 125, 250, 500, and 1000 Hz were associated with the incidence of hyperglycaemia (all p values <0.050), with the highest risk of 1.27 (95% CI: 1.10, 1.47) at 31.5 Hz (p = 0.001). The association between exposure to occupational noise and incident hyperglycaemia was modified by sex, as shown in Supplemental Table S2 (p = 0.023). A 5-dBA increase in occupational noise was associated with a 1.49-fold increase in the risk of incident hyperglycaemia among women (95% CI: 1.13, 1.97; p = 0.005).

## Discussion

Subjects exposed to occupational noise levels ≥ 85 dBA had a significantly higher risk of hyperglycaemia than those exposed to < 70 dBA. We also observed a significant exposure-response relationship among the high-, medium-, and low-exposure groups (ARR = 1.33; 95% CI: 1.02, 1.73). These results are consistent with findings of a population-based cohort study of 57,053 residents, which found a significant and increased risk of incident diabetes (ARR = 1.11; 95% CI: 1.03, 1.19) per 10-dB increase in road traffic noise^21^, and with findings of a case-crossover study in Madrid (Spain) (2001–2009), which reported a strong association between a rise of 0.5 dBA in night-time traffic noise at a 1-day lag and a 4.6% risk (95% CI: 1.5, 7.8) of diabetic mortality^22^. The present and previous two studies all indicate the possibility that noise-induced CVD may result from the activation of impaired metabolism, which leads to increased blood glucose levels^12,13^. In contrast, a cross-sectional survey did not show a significant association between self-reported occupational noise exposure and diabetes among 23,486 European participants^25^. The inconsistency in the findings may be due to the more accurate exposure assessment for the measured and modelled noise levels^21,22^ than for the reported subjective ones^25^. In addition to noise-induced hearing loss^26,27^ and CVD^1–10^, the possibility of hyperglycaemia from occupational noise exposure should be considered in future studies.

A sensitivity analysis was also conducted to elucidate the association between occupational noise exposure and incident diabetes. A higher but not significant risk of diabetes (ARR = 3.31, 95% CI: 0.69, 15.95; p = 0.136) was observed in workers exposed to ≥ 85 dBA than in those exposed to < 70 dBA due to the small number of diabetes cases (n = 15) identified only by the measurement of FBG level ≥ 126 mg/dL.

Exposure to high noise levels at 31.5, 63, 125, 250, 500, 1000, and 2000 Hz was associated with the incidence of hyperglycaemia, and the highest risk was found at 31.5 Hz. Significant exposure-response relationships among high-, medium- and low-exposure groups for incident hyperglycaemia were also identified at 31.5 Hz, 63 Hz, 125 Hz, 250 Hz, 500 Hz, 1000 Hz, and 2000 Hz. These findings indicated that machinery and equipment manufacturing workers might be more sensitive to low and medium frequencies, leading to elevated blood glucose. The real reason for such observations is unknown. A cross-sectional study also reported a strong association between diabetes and hearing loss at low- and medium-frequencies^28^. Annoyance and stress caused by the low frequency of noise at work might be the other reason for the increased risk of diabetes^24,29^. Therefore, we recommend conducting experimental or animal studies in the future to investigate the association between the frequency spectrum of noise exposure and pathophysiological functions.

We observed that 5-dB increases at frequencies of 31.5, 63, 125, 250, 500, and 1000 Hz were associated with an increased risk of hyperglycaemia, which may indicate that noise-induced hyperglycaemia involves multiple pathways among the various biological mechanisms. Low-frequency occupational noise exposure (i.e., 31.5, 63, 125, and 250 Hz) may pose a risk of hyperglycaemia indirectly through responses such as annoyance and the disturbance experienced during activities requiring selective attention or while dealing with a high load of information^12,13,30–32^. In contrast, middle-frequency occupational noise exposure (i.e., 500 and 1000 Hz) may cause hyperglycaemia directly by repeated and prolonged stimulation of the autonomic nervous and endocrine systems^12,13,33,34^. However, more evidence is required to elucidate the reasons for the association between frequency components of noise exposure and the incidence of hyperglycaemia.

We also calculated the cumulative noise exposure in dB-years based on the equation used in a previous study^8^ to account for the subjects’ duration of exposure. Subjects were classified as low-exposure (officers; cumulative noise levels: 77.0 ± 5.4 dBA-years), medium-exposure (< 95 dBA-years; cumulative noise levels: 84.6 ± 7.6 dBA-years), and high-exposure (≥ 95 dBA-years; cumulative noise levels: 99.0 ± 3.8 dBA-years) groups. We found that the high-exposure (ARR = 1.62; 95% CI: 0.96, 2.76; p = 0.074) and medium-exposure groups (ARR = 1.32; 95% CI: 0.86, 2.03; p = 0.202) had an increased risk of hyperglycaemia compared with the low-exposure group, but the results were not significant after adjusting for age, sex, triglyceride level, hypertension, family history of diabetes, and the use of hearing protection devices. The exposure-response association between cumulative noise exposure and the risk of hyperglycaemia was marginally significant (ARR = 1.28; 95% CI: 0.99, 1.65; p = 0.055).

From a psychoacoustic perspective, the human response to sound is contingent on both exposure intensity and the frequency characteristics of the stimulus. However, our results do not show an interaction of personal noise levels and frequency components, indicating the association between occupational noise exposure and incident hyperglycaemia mainly due to the stress reaction from the auditory stimuli.

The association between occupational noise exposure and incident hyperglycaemia was significantly influenced by sex in the present study. Women had a higher risk of hyperglycaemia than men. One previous study reported no significant effect modification by sex but did find a stronger relationship between hyperglycaemia and road traffic noise among women (ARR = 1.11; 95% CI: 1.03, 1.20) than among men (ARR = 1.05; 95% CI: 0.98, 1.13)^21^. This modifier should be considered when investigating the association between noise exposure and hyperglycaemia in future studies.

The strength of this study lies in its retrospective cohort design, which was formulated to calculate the observed person-years and distinguish the noise-induced effect after a longitudinal follow-up. This design provides incident hyperglycaemia instead of prevalent hyperglycaemia in the cross-sectional study to investigate temporal associations with occupational noise exposure. In addition, personal exposure assessment, environmental noise measurements, and octave-band analyses of workstaion noise were conducted to provide a precise and accurate evaluation of noise exposure in a real-world workplace setting.

This study has many limitations that must be mentioned. The major restriction of the retrospective design is the generation of a healthy-worker effect that may produce a lower proportion of hyperglycaemia cases in the high noise-exposure group or a higher incidence of hyperglycaemia in the low noise-exposure group. Such a design also restricts the establishment of a job-exposure matrix to provide quantitative exposure-response and noise-frequency characteristics analyses for subjects. The second limitation is the underestimation of the observed person-years because of missing information of noise exposure history before the subjects were employed with the current company or before the diagnosis of diabetes by a physician. The third limitation is the lack of collection of data on noise exposure outside the workplace during the employment period. Exposure to aircraft and road traffic noise has been reported with an increased risk of diabetes in a meta-analysis^24^. The fourth limitation is the potential recall bias of lifestyle habits that affect diabetes, which was only measured for 2012. The fifth limitation is that other co-exposure factors (i.e., particulate matter or gaseous pollutants) in the workplace, which could be correlated with noise, were not considered. Sixth, we did not control for socioeconomic status in the Cox regression models, although educational levels (often used as proxy of socioeconomic status) were not significantly associated with incident hyperglycaemia in this study. Seventh, we only measured fasting glucose levels but not post-prandial glucose levels. The ideal requirement would be to provide both measurements. Eighth, the lack of physiological measurements of stress hormones (such as cortisol, epinephrine or norepinephrine, etc.) and autonomic function tests to measure sympathetic over-activity before the study began, during the study period, and at the end of the study restricts the significance of the evidence for noise exposure as a causative factor of hyperglycaemia. Ninth, the subjects’ information about sleep patterns and noise annoyances at home was not collected. Noise-induced sleep disturbances and annoyances are both possible mechanisms that could generate cardio-metabolic effects^24^, and we could not rule out sleep disturbances as a contributing factor. Tenth, we did not evaluate the psychological factors that may induce stress as a causative factor of incident hyperglycaemia. Finally, we did not measure fasting glucose from the start of the study until the end, which makes it difficult to determine how and when the effect of noise pollution could have led to hyperglycaemia. Therefore, the association between noise pollution and incident hyperglycaemia cannot be elaborated as being that of causality.

## Conclusions

In summary, this study observed an association between occupational noise exposure and an increased risk of incident hyperglycaemia. Positive and linear exposure–response relationships were demonstrated for noise frequency components at 31.5, 63, 125, 250, 500, 1000, and 2000 Hz. Machinery and equipment manufacturing workers who are exposed to noise levels at 31.5 Hz may have the greatest risk of hyperglycaemia. These findings provide a possible link between noise exposure and cardio-metabolic disease. We recommend future studies be conducted to determine the associations between hyperglycaemia and octave-band frequencies of occupational noise exposure in different industries.

## Methods

### Study population

The detailed procedures for inviting companies to cooperate were mentioned in a previous study^10^. Briefly, we recruited 1028 volunteers from four machinery and equipment manufacturing companies in 2012. Among them, two subjects with a history of diabetes before employment and 121 subjects who were followed-up for less than one year were excluded. Finally, we enrolled 905 study subjects in this industry-based cohort. Workers exposed to high noise levels in the processes of metal cutting, pressing, grinding, sand blasting, polishing, and gear washing. No subjects reported having shift work.

The Institutional Review Board of the School of Public Health, China Medical University reviewed and approved this study. Written informed consent was acquired from each participant. Additionally, all methods were performed in accordance with the relevant guidelines and regulations.

### Patients with hyperglycaemia

We required all participants to fast overnight before blood sampling during the annual health examinations performed in 2012. Venous blood samples collected by trained nurses were used to perform blood glucose measurements by a standard glucose oxidase method. Hyperglycaemia was defined as a positive response to either “have you been diagnosed with prediabetes by a physician?” or “did you start using hypoglycaemic drugs after your employment start date in the current company?” or if upon assessment in 2012, the FBG level was ≥ 100 mg/dL^35^. In addition, height, body weight, SBP, diastolic blood pressure (DBP), total cholesterol level, high-density lipoprotein cholesterol, low-density lipoprotein cholesterol, and triglyceride levels were measured for all subjects. A trained nurse applied an automated sphygmomanometer (Ostar model P2; Ostar Meditech Corp., Taipei, Taiwan) to measure each subject’s bilateral blood pressure in the sitting position, and the mean of two measurements was used to represent an individual’s blood pressure. We defined hypertensive patients as those with one or more of the following criteria: a diagnosis of hypertension by a physician; the taking of antihypertensive medicine; an SBP of ≥ 140 mmHg; or a DBP of ≥ 90 mmHg.

Potential risk factors related to hyperglycaemia or diabetes were identified with a self-administered questionnaire. Demographic characteristics, lifestyle habits, family history of diabetes, working activity, and the use of hearing-protection devices were collected and defined specifically to avoid information bias^10,36^. Working activity was considered as each subject’s duration of sitting, walking, lifting heavy objects during working periods, and the distance walked between the workplace and home (which was further categorized into high and low levels based on the cut-off point of 10 in a scoring system)^37^.

### Follow-up

We collected the date of first employment for each subject based on employment personnel records obtained from the four companies and assigned this date retrospectively as the starting time for the follow-up. The end of the follow-up period was established as either the date that the patient was diagnosed with prediabetes by a physician, the date of hypoglycaemic medication initiation, or the date at which blood glucose was measured in December 2012.

### Occupational noise exposure evaluation and frequency component analyses

The processes of noise exposure evaluation and frequency component analyses were described in detail in a previous study^10^. Briefly, we conducted a walk-through survey and combined the workplace information to identify different numbers of similar exposure groups (SEGs) for each participating company. Workers assigned to the same SEGs showed similarities in the types and frequency of tasks, agents, and processes involved and in the way of performing tasks^38^.

We used a personal noise dosimeter (Logging Noise Dose Metre Type 4443, Brüel & Kjær, Nærum, Denmark) to automatically report 5-minute continuous equivalent sound levels (Leq) in dBA during working periods (8:00–17:00). A total of 96 5-minute Leq values (excluding those obtained from 12:00–13:00 pm) was used to calculate one 8-h time-weighted average (TWA) noise level for each SEG. Before conducting noise measurements, we calibrated this dosimeter with a sound-level calibrator (Type 4231, Brüel & Kjær, Nærum, Denmark) and set up its determining range between 50–120 dBA for all SEGs.

In addition, an octave-band analyser (TES-1358, TES Electronic Corp., Taipei, Taiwan) was used to record the 5-minute continuous Leq in decibels (dB) at frequencies of 31.5, 63, 125, 250, 500, 1000, 2000, 4000, and 8000 Hz during the monitoring periods. We applied all 96 5-minute Leq values at each frequency to calculate one 8-h TWA noise level for a specific frequency component. The analyser was calibrated by a sound-level calibrator (TES-1356, TES Electronic Corp., Taipei, Taiwan) prior to the noise measurements. The 8-h TWA Leq and its octave-band frequencies were collected by two occupational hygienists to allocate specific levels of environmental noise (dBA) and octave-band frequencies (dB) for each SEG in the four companies.

Because the regulatory workplace monitoring in these companies showed no significant difference in noise levels within the last 10 years (82.8 ± 9.1 dBA vs 82.0 ± 8.3 dBA) and personal levels of noise exposure and frequency components were not available to classify subjects into different exposure groups, we assumed that the personal noise levels and the frequency spectrum of the occupational noise were equal and stable over the follow-up period. Using the cut-off value of 85 dBA, subjects were divided into high-exposure (field workers exposed to ≥ the median (85 dBA)), medium-exposure (field workers exposed to < 85 dBA), and low-exposure (office workers) groups, based on the noise exposure assessment, using a statistical approach. The cut-off value of 85 dBA is an important limit for occupational safety legislation and regulations and is often adopted in other studies^9,11^. The classification into groups was due to the large variations in median noise levels and exposure ranges at the nine frequencies, as shown in Supplemental Figure S1. Additionally, we used a 5-dBA increase in personal noise exposure and a 5-dB increase in octave-band analyses of workstation noise to investigate associations with hyperglycaemia.

### Statistical analyses

The Shapiro-Wilk test was used to determine the normality of continuous variables. The Kruskal-Wallis test was used to perform multiple comparisons of continuous variables with non-normal data distributions between the three groups. We also applied the chi-square test and Wilcoxon rank-sum test to identify differences in dichotomous and continuous variables among the three groups, respectively. In addition, non-parametric Spearman correlation coefficients were estimated to investigate the correlation between individual noise exposure, workstation noise levels, and octave-band frequencies of workstation noise in the workplace.

Because this retrospective cohort study only obtained one measurement of the FBG in December 2012, each participant’s baseline FBG was measured at the time of employment. The average length of time between baseline non-hyperglycaemia FBG and follow-up FBG measurements was 7.5 years (median: 5.3 years; IQR: 7.9 years). We summed the number of hyperglycaemia patients identified by the questionnaire (n = 1) or by FBG measurements (n = 118) as the health outcome to conduct the Cox proportional hazard regression analyses. The RRs with 95% CIs were calculated to compare differences in incident hyperglycaemia among the different groups. We used the change-in-estimate method to identify covariates in the best fit model^39^. First, all candidate variables were individually added to the basic model with the exposure variable and outcome for finding one variable with the largest change in the estimated exposure effect. The exposure variable combined with this variable (i.e., the use of personal protective equipment) was built as the Model 1 to estimate the risk of incident hyperglycaemia. The adjustment for the use of hearing-protection devices in the model can avoid exposure bias^40,41^. After that, every possible combination of remaining variables (i.e., age, sex, BMI, employment duration, educational level, SBP, DBP, total cholesterol, triglyceride level, cigarette smoking, alcohol, tea and coffee consumption, regular exercise, hypertension, family history of diabetes, and working activity) was added to the Model 1 to see if a 10% change in the RR of the exposure variable until no more input of variables to exceed this criteria^39^. Two variables of hypertension and triglyceride level combined with the Model 1 were identified to generate a 10% increase in the RR of the exposure variable as the Model 2. Model selection was repeated for every combination of exposure variables (i.e., high- and medium- vs. low-exposure groups or continuous per 5-dB increase) and the outcome to ensure similar selections of covariates. Finally, we added two variables of age and sex to present the biological plausibility and one variable of a family history of diabetes to account for genetic effects^42,43^ in creating the final model (i.e., Model 3). The step to select a priori important variables into the model can avoid overfitting^39,44^. We did not include the employment duration in the final model due to its high correlation with age (Spearman’s correlation coefficient: 0.648, p < 0.001). The guideline of 10 to 15 observations per predictor is suggested to produce the reasonably stable estimates in the survival models^44^. Because there were 119 hyperglycaemia patients in this study, it was enough to support 6 covariates with the exposure variable in the final model against overfitting. The proportionality assumption was tested by including time-dependent covariates in the Cox model. Six time-dependent covariates were generated by creating interactions with the predictors, including age, sex, triglyceride levels, hypertension, family history of diabetes, and the use of hearing protection devices. None of the time-dependent covariates were significant (all p values > 0.050), indicating that the assumptions of the Cox models were satisfied. Additionally, a random intercept with a log-normal distribution was specified to test the multilevel nature of our data, and the non-significant result (p = 0.459) indicated that no effects of sampling clusters (the companies) were present in this study.

We also established two separate Cox regression models, one with a linear noise-exposure term (linear model) and another with a squared noise-exposure term (nonlinear model), to test the exposure-response relationship after adjusting for potential confounders and effect modifiers. We compared the Akaike information criterion (AIC) value of the linear model with that of the nonlinear model. The AIC is a likelihood-based model selection statistic, with a lower value indicating a better fit of the underlying data. Because the AIC value of the linear model (1259.592) was smaller than that of the nonlinear model (1261.551), the exposure-response relationship was fitted better for linearity.

Stratified analyses were used to determine the effect modification of selected demographic characteristics and to test the interaction between the high-exposure and low-exposure groups. The interactions were tested at a significance level of p < 0.050. In addition, the interaction between personal noise levels and specific frequency components was evaluated in the Cox regression models. We applied the SAS standard package for Windows version 9.4 (SAS Institute Incorporation, Cary, North Carolina, USA) to analyse the data and set the significance level at 0.050 for all two-tailed tests.

### Ethical approval and consent to participate

All participants provided informed consent, and the protocol was reviewed and approved by the Institutional Review Board of the School of Public Health, China Medical University (No. 100-03-10-4).

## Supplementary information

Supplemental Material.

## Data Availability

The datasets generated and analysed during the current study are not publicly available due the confidentiality agreement with participating companies but are available from the corresponding author on reasonable request.

## References

[1] McNamee R, Burgess G, Dippnall WM, Cherry N (2006). Occupational noise exposure and ischaemic heart disease mortality. Occup. Environ. Med..

[2] Virkkunen H, Kauppinen T, Tenkanen L (2005). Long-term effect of occupational noise on the risk of coronary heart disease. Scand. J. Work. Environ. Health.

[3] Virkkunen H, Harma M, Kauppinen T, Tenkanen L (2006). The triad of shift work, occupational noise, and physical workload and risk of coronary heart disease. Occup. Environ. Med..

[4] Gan WQ, Davies HW, Demers PA (2011). Exposure to occupational noise and cardiovascular disease in the United States: the National Health and Nutrition Examination Survey 1999-2004. Occup. Environ. Med..

[5] Davies HW (2005). Occupational exposure to noise and mortality from acute myocardial infarction. Epidemiology.

[6] Willich SN, Wegscheider K, Stallmann M, Keil T (2006). Noise burden and the risk of myocardial infarction. Eur. Heart J..

[7] Bortkiewicz A (2010). Work-related risk factors of myocardial infarction. Int. J. Occup. Med. Environ. Health.

[8] Sbihi H, Davies HW, Demers PA (2008). Hypertension in noise-exposed sawmill workers: a cohort study. Occup. Environ. Med..

[9] Chang TY (2013). Occupational noise exposure and incident hypertension in men: a prospective cohort study. Am. J. Epidemiol..

[10] Liu CS, Young LH, Yu TY, Bao BY, Chang TY (2016). Occupational Noise Frequencies and the Incidence of Hypertension in a Retrospective Cohort Study. Am. J. Epidemiol..

[11] Teixeira LR (2019). WHO/ILO work-related burden of disease and injury: Protocol for systematic reviews of exposure to occupational noise and of the effect of exposure to occupational noise on cardiovascular disease. Environ. Int..

[12] Babisch W (2002). The Noise/Stress Concept, Risk Assessment and Research Needs. Noise Health.

[13] Munzel T, Gori T, Babisch W, Basner M (2014). Cardiovascular effects of environmental noise exposure. Eur. Heart J..

[14] Recio A, Linares C, Banegas JR, Diaz J (2016). Road traffic noise effects on cardiovascular, respiratory, and metabolic health: An integrative model of biological mechanisms. Environ. Res..

[15] Wu HP, Cheng TJ, Tan CT, Guo YL, Hsu CJ (2009). Diabetes impairs recovery from noise-induced temporary hearing loss. Laryngoscope.

[16] Wu HP, Hsu CJ, Cheng TJ, Guo YL (2010). N-acetylcysteine attenuates noise-induced permanent hearing loss in diabetic rats. Hear. Res..

[17] Cui B, Gai Z, She X, Wang R, Xi Z (2016). Effects of chronic noise on glucose metabolism and gut microbiota-host inflammatory homeostasis in rats. Sci. Rep..

[18] Liu L (2018). Chronic noise-exposure exacerbates insulin resistance and promotes the manifestations of the type 2 diabetes in a high-fat diet mouse model. PLoS one.

[19] Jang TW, Kim BG, Kwon YJ, Im HJ (2011). The association between impaired fasting glucose and noise-induced hearing loss. J. Occup. Health.

[20] Dzhambov AM, Dimitrova DD (2016). Long-term self-reported exposure to occupational noise is associated with BMI-defined obesity in the US general population. Am. J. Ind. Med..

[21] Sorensen M (2013). Long-term exposure to road traffic noise and incident diabetes: a cohort study. Environ. Health Perspect..

[22] Tobias A, Diaz J, Recio A, Linares C (2015). Traffic noise and risk of mortality from diabetes. Acta Diabetol..

[23] Cai Y (2017). Long-term exposure to road traffic noise, ambient air pollution, and cardiovascular risk factors in the HUNT and lifelines cohorts. Eur. Heart J..

[24] Zare Sakhvidi MJ, Zare Sakhvidi F, Mehrparvar AH, Foraster M, Dadvand P (2018). Association between noise exposure and diabetes: A systematic review and meta-analysis. Environ. Res..

[25] Dzhambov AM (2017). Exposure to self-reported occupational noise and diabetes - A cross-sectional relationship in 7th European Social Survey (ESS7, 2014). Int. J. Occup. Med. Environ. Health.

[26] Rubak T, Kock SA, Koefoed-Nielsen B, Bonde JP, Kolstad HA (2006). The risk of noise-induced hearing loss in the Danish workforce. Noise Health.

[27] Seixas NS (2012). 10-Year prospective study of noise exposure and hearing damage among construction workers. Occup. Environ. Med..

[28] Bamanie AH, Al-Noury KI (2011). Prevalence of hearing loss among Saudi type 2 diabetic patients. Saudi Med. J..

[29] Leventhall HG (2004). Low frequency noise and annoyance. Noise Health.

[30] Pawlaczyk-Luszczynska M, Dudarewicz A, Waszkowska M, Sliwinska-Kowalska M (2003). Assessment of annoyance from low frequency and broadband noises. Int. J. Occup. Med. Environ. Health.

[31] Bengtsson J, Waye KP, Kjellberg A (2004). Evaluations of effects due to low-frequency noise in a low demanding work situation. J. Sound. Vib..

[32] Pawlaczyk-Luszczyniska M, Dudarewicz A, Waszkowska M, Szymczak W, Sliwinska-Kowalska M (2005). The impact of low-frequency noise on human mental performance. Int. J. Occup. Med. Environ. Health.

[33] Truswell WHT, Randolph KJ, Snyder GG (1979). The effect of static tympanic pressure gradients on hearing sensitivity in normal subjects. Laryngoscope.

[34] Maurizi M, Paludetti G, Ottaviani F, Rosignoli M (1984). Auditory brainstem responses to middle- and low-frequency tone pips. Audiology.

[35] American Diabetes A (2013). Standards of medical care in diabetes–2013. Diabetes care.

[36] Chang TY (2012). Noise frequency components and the prevalence of hypertension in workers. Sci. Total. Environ..

[37] Hwang LC, Chen CJ, Tsieng WP (1997). A nested case control study on multiple risk factors for acute fatal cerebrovascular accident and coronary heart disease. Chin. J. Fam. Med..

[38] Nulhausen, J. R. & Damiano, J. *A strategy for assessing and managing occupational exposure*. Second edn, (American Industrial Hygiene Association, 1998).

[39] Greenland S (1989). Modeling and variable selection in epidemiologic analysis. Am. J. Public. Health.

[40] Armstrong BG (1998). Effect of measurement error on epidemiological studies of environmental and occupational exposures. Occup. Environ. Med..

[41] Sbihi H, Teschke K, MacNab YC, Davies HW (2010). An investigation of the adjustment of retrospective noise exposure for use of hearing protection devices. Ann. Occup. Hyg..

[42] Uusitupa MI (2011). Impact of positive family history and genetic risk variants on the incidence of diabetes: the Finnish Diabetes Prevention Study. Diabetes care.

[43] Rojo-Martinez G (2020). Incidence of diabetes mellitus in Spain as results of the nation-wide cohort di@bet.es study. Sci. Rep..

[44] Babyak MA (2004). What you see may not be what you get: a brief, nontechnical introduction to overfitting in regression-type models. Psychosom. Med..

