# Supplementary material for: Occupational noise exposure and its association with incident hyperglycaemia: a retrospective cohort study
Source: Sci Rep. 2020 May 22;10:8584. doi: 10.1038/s41598-020-65646-1 (PMC7244742; doi:10.1038/s41598-020-65646-1)
Supplement: Supplementary file 1 — Supplemental Material. [file 41598_2020_65646_MOESM1_ESM.docx]

**SUPPLEMENTAL MATERIAL**

**Occupational noise exposure and its association with incident hyperglycaemia: a retrospective cohort study**

Ta-Yuan Chang ^1*^, Tzu-Yi Yu ^1^, Chiu-Shong Liu ^2^, Li-Hao Young ^1^, Bo-Ying Bao ^3,4^

^1^ Department of Occupational Safety and Health, College of Public Health, China Medical University, No. 91, Hsueh-Shih Road, Taichung 40402, Taiwan, Republic of China.

^2^ Department of Family Medicine, China Medical University Hospital, No. 2, Yuh-Der Road, Taichung 40447, Taiwan, Republic of China.

^3^ Department of Pharmacy, College of Pharmacy, China Medical University, No. 91, Hsueh-Shih Road, Taichung 40402, Taiwan, Republic of China.

^4^ Department of Nursing, Asia University, No. 500, Lioufeng Road, Wufeng, Taichung 41354, Taiwan, Republic of China.

Email addresses: TYC – [tychang@mail.cmu.edu.tw](mailto:tychang@mail.cmu.edu.tw); TYY – [fish_ever_022204@hotmail.com](http://portal.cmu.edu.tw/eip/addressQuery.do?id=1337775619445); CSL – [liucs@ms14.hinet.net](mailto:liucs@ms14.hinet.net); LHY – [lhy@mail.cmu.edu.tw](mailto:lhy@mail.cmu.edu.tw); BYB – [bao@mail.cmu.edu.tw](mailto:bao@mail.cmu.edu.tw);

^*^ Corresponding author

**Table of contents:** Page

_____________________________________________________________________**TABLES**

[Supplemental Table S 1. Spearman’s ρ correlations of occupational noise between personal levels (dBA), workstation levels (dBA), and specific frequency components (dB) in Taichung, Taiwan. 3](#_Toc33302681)

[Supplemental Table S 2. Modification of association between incident hyperglycaemia and occupational noise (5-dBA increase) by demographic characteristics among 905 participants in Taichung, Taiwan. 4](#_Toc33302682)

FIGURES

[Supplemental Figure S 1. Distributions of occupational noise (LAeq) for personal exposure (dBA), workstation levels (dBA), and different frequency components (dB) among participants measured in 2012. 5](#_Toc33302757)

[Supplemental Figure S 2. Adjusted relative risk (ARR)^a^ of hyperglycaemia for the high-exposure group compared with the low-exposure group stratified by appropriate demographic characteristics. 6](#_Toc33302758)

**Supplemental Table S 1.** Spearman’s ρ correlations of occupational noise between personal levels (dBA), workstation levels (dBA), and specific frequency components (dB) in Taichung, Taiwan.

| **Noise types** | **Number** | **Correlation coefficients** | | | | | | | | | | |
| --- | --- | --- | --- | --- | --- | --- | --- | --- | --- | --- | --- | --- |
|  |  | **Personal** | **Workstation** | **Low frequency (Hz)** | | | **Medium frequency (Hz)** | | | **High frequency (Hz)** | | |
|  |  |  |  | **31.5** | **63** | **125** | **250** | **500** | **1000** | **2000** | **4000** | **8000** |
| Personal | 905 | 1 | 0.833^a^ | 0.717^a^ | 0.712^a^ | 0.783^a^ | 0.814^a^ | 0.839^a^ | 0.833^a^ | 0.795^a^ | 0.771^a^ | 0.766^a^ |
| Workstation | 905 | - | 1 | 0.834^a^ | 0.867^a^ | 0.942^a^ | 0.943^a^ | 0.945^a^ | 0.965^a^ | 0.861^a^ | 0.786^a^ | 0.780^a^ |
| 31.5 Hz | 905 | - | -  - | 1 | 0.889^a^ | 0.889^a^ | 0.863^a^ | 0.850^a^ | 0.816^a^ | 0.680^a^ | 0.564^a^ | 0.550^a^ |
| 63 Hz | 905 | - | -  - | - | 1 | 0.927^a^ | 0.907^a^ | 0.896^a^ | 0.861^a^ | 0.781^a^ | 0.666^a^ | 0.647^a^ |
| 125 Hz | 905 | - | -  - | - | - | 1 | 0.982^a^ | 0.966^a^ | 0.946^a^ | 0.822^a^ | 0.718^a^ | 0.706^a^ |
| 250 Hz | 905 | - | -  - |  | - |  | 1 | 0.972^a^ | 0.954^a^ | 0.833^a^ | 0.741^a^ | 0.738^a^ |
| 500 Hz | 905 | - | -  - |  | - |  | - | 1 | 0.971^a^ | 0.847^a^ | 0.738^a^ | 0.718^a^ |
| 1000 Hz | 905 | - | -  - |  | - |  | - | - | 1 | 0.879^a^ | 0.774^a^ | 0.756^a^ |
| 2000 Hz | 905 | - | -  - |  | - |  | - | - | - | 1 | 0.929^a^ | 0.869^a^ |
| 4000 Hz | 905 | - | -  - |  | - |  | - | - | - | - | 1 | 0.967^a^ |
| 8000 Hz | 905 | - | -  - |  | - |  | - | - | - | - | - | 1 |

dB, decibel; dBA, A-weighted decibel. ^a^*P* < 0.05.

**Supplemental Table S 2.** Modification of association between incident hyperglycaemia and occupational noise (5-dBA increase) by demographic characteristics among 905 participants in Taichung, Taiwan.

| Covariate |  | N=905 | Incidence | ARR^a^ (95% CI) | *P* value | *P* value for interaction |
| --- | --- | --- | --- | --- | --- | --- |
| Sex | Male | 726 | 98/5220=0.0188 | 1.05 (0.92, 1.19) | 0.487 | 0.023 |
|  | Female | 179 | 21/1591=0.0132 | 1.49 (1.13, 1.97) | 0.005 |  |
| Age | ≥36 years | 458 | 85/4855=0.0175 | 1.13 (0.99, 1.29) | 0.083 | 0.632 |
|  | <36 years | 447 | 34/1956=0.0174 | 1.08 (0.87, 1.33) | 0.493 |  |
| Triglyceride level | ≥99 mg/dl | 457 | 81/3675=0.0220 | 1.08 (0.94, 1.23) | 0.306 | 0.545 |
|  | <99 mg/dl | 448 | 38/3136=0.0121 | 1.21 (0.98, 1.48) | 0.073 |  |
| Hypertension | Yes | 238 | 49/1914=0.0256 | 1.08 (0.89, 1.31) | 0.443 | 0.900 |
|  | No | 667 | 70/4897=0.0143 | 1.13 (0.98, 1.30) | 0.107 |  |
| Family history of | Yes | 175 | 30/1504=0.0199 | 1.15 (0.89, 1.48) | 0.282 | 0.788 |
| diabetes | No | 730 | 89/5307=0.0168 | 1.11 (0.98, 1.27) | 0.111 |  |
| Use of hearing- | Yes | 44 | 4/417=0.0096 | 3.10 (0.74, 13.00) | 0.122 | 0.934 |
| protection devices | No | 861 | 115/6394=0.0180 | 1.12 (0.99, 1.25) | 0.064 |  |

ARR, adjusted relative risk; CI, confidence interval.

^a^ Cox proportional hazards regression adjusting for age, sex, hypertension, triglyceride level, family history of diabetes, and the use of hearing-protection devices without the modifying factor in each model.

**Supplemental Figure S 1.** Distributions of occupational noise (LAeq) for personal exposure (dBA), workstation levels (dBA), and different frequency components (dB) among participants measured in 2012.

dB, decibel; dBA, A-weighted decibel; LAeq, A-weighted equivalent sound level.


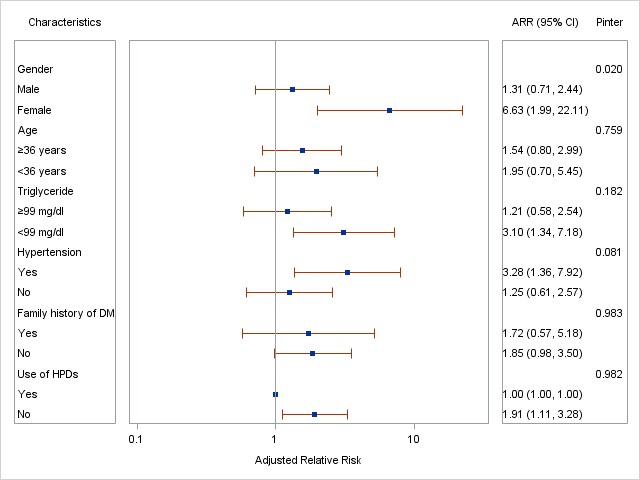


**Supplemental Figure S 2.** Adjusted relative risk (ARR)^a^ of hyperglycaemia for the high-exposure group compared with the low-exposure group stratified by appropriate demographic characteristics.

ARR, adjusted relative risk; CI, confidence interval; DM, diabetes Mellitus; HPDs, hearing-protection devices. ^a^ Cox proportional hazards regression adjusted for age, sex, hypertension, triglyceride level, family history of DM, and the use of HPDs when appropriate.
